# An evaluation of a recombinant multiepitope based antigen for detection of *Toxoplasma gondii* specific antibodies

**DOI:** 10.1186/s12879-017-2920-9

**Published:** 2017-12-29

**Authors:** Khalid Hajissa, Robaiza Zakaria, Rapeah Suppian, Zeehaida Mohamed

**Affiliations:** 1 Department of Zoology, Faculty of Science and Technology, Omdurman Islamic University, B.O.Box, 382 Omdurman, Sudan; 2 Department of Medical Microbiology & Parasitology, School of Medical Sciences, Universiti Sains Malaysia, 16150 Kubang Kerian, Kelantan Malaysia; 3 Biomedicine Program, School of Health Sciences, Universiti Sains Malaysia, 16150 Kubang Kerian, Kelantan Malaysia

**Keywords:** *T. Gondii*, Elisa, Multiepitope, USM.TOXO1, Serodiagnosis, Recombinant antigens

## Abstract

**Background:**

The inefficiency of the current tachyzoite antigen-based serological assays for the serodiagnosis of *Toxoplasma gondii* infection mandates the need for acquirement of reliable and standard diagnostic reagents. Recently, epitope-based antigens have emerged as an alternative diagnostic marker for the achievement of highly sensitive and specific capture antigens. In this study, the diagnostic utility of a recombinant multiepitope antigen (USM.TOXO1) for the serodiagnosis of human toxoplasmosis was evaluated.

**Methods:**

An indirect enzyme-linked immunosorbent assay (ELISA) was developed to evaluate the usefulness of USM.TOXO1 antigen for the detection of IgG antibodies against *Toxoplasma gondii* in human sera. Whereas the reactivity of the developed antigen against IgM antibody was evaluated by western blot and Dot enzyme immunoassay (dot-EIA) analysis.

**Results:**

The diagnostic performance of the new antigens in IgG ELISA was achieved at the maximum values of 85.43% and 81.25% for diagnostic sensitivity and specificity respectively. The USM.TOXO1 was also proven to be reactive with anti- *T. gondii* IgM antibody.

**Conclusions:**

This finding makes the USM.TOXO1 antigen an attractive candidate for improving the toxoplasmosis serodiagnosis and demonstrates that multiepitope antigens could be a potential and promising diagnostic marker for the development of high sensitive and accurate assays.

## Background

*Toxoplasma gondii* (*T. gondii*) is a widely distributed intercellular parasite with a relatively wide host range including human and almost all warm-blooded animals [1]. The clinical complications of the disease, especially in immunocompromised patients emphasize the importance of accurately identifying the infection. In particular, early diagnosis is critical for the effective therapy of the disease [2]. The important role of accurate diagnosis for the clinical management of toxoplamososis is a public health concern [3]. To date, various diagnostic techniques have been established [4]. However, the routine diagnostic strategy is mainly based on the detection of *T. gondii*-specific antibodies by various serological tests [5]. The serological tests play a vital role in the diagnosis of both human and animal toxoplasmosis [6].

Despite the satisfactory results obtained from the serodiagnosis specifically ELISA, development of standard and reliable reagents remains laborious and expensive [7, 8]. Furthermore, the insufficient accuracy of several serodiagnostic tests necessitates the exploration of alternative reagents to be used for diagnostic purposes in the progress of toxoplasmosis control [8, 9]. On the basis of this, suggestions were put forward to identify possible future directions of research on the development of accurate diagnostic tests. The scientific response to this scenario was based on paying particular attention to the recombinant multiepitope antigens that express different immunoreactive regions of various *T. gondii* antigens [10].

Recently, epitope based antigen has emerged as alternative tools for achievement of highly sensitive and specific capture antigens, that can be used as an alternative source of antigens with the potential to successfully replace the native antigen [10, 11]. The rationale behind using of epitope based antigen for improvement of toxoplasmosis serodiagnosis would prove highly beneficial to increase the sensitivity and specificity; thus improve the standardization of the tests [12]. Furthermore, the more advantages of using such kind of antigens, is the capture antigens composition are precisely known and therefore mixture of different antigens can be used, as well as the cost of antigens production can be significantly reduced [12]. Such reasons justify why the studies on the *T. gondii* epitope antigens are receiving increasing attention from researchers.

The use of epitope-based antigen for the development of new diagnostic tests of various infections has shown encouraging results against various diseases. These diseases include hepatitis C virus [13], leishmaniasis [14], trypanosomiasis [15], leprosy [16], leptospirosis and *Mycobacterium tuberculosis* [17, 18], as well as toxoplasmosis [8, 19, 20]. The advancement in bioinformatics and synthetic biology provides alternative strategies toward novel design and production of such kind of antigens [21]. These approaches are allowing the design and the subsequent synthesis of recombinant protein with improved or novel antigenic characteristics and reduced production costs [22]. Thus, studies on *T. gondii* multi-epitope antigens are presently gaining increasing attention. This approach was adopted in the present study to generate a single multiepitope-based antigen expressing nine potential immunodominant epitopes of *T. gondii*. Consequently, the accuracy of the entire protein as a diagnostic marker for toxoplasmosis in humans was investigated.

## Methods

### Serum samples

Hospital Universiti Sains Malaysia (HUSM) of Kelantan is situated at the north east of peninsular Malaysia. It is a 700-beded tertiary teaching hospital for undergraduate medical program and postgraduate master of medicine. The hospital was equipped with accredited laboratories for testing all clinical samples from patients, including the Microbiology laboratory. A total of 247 human serum samples were collected from patients requested for routine serological investigation for toxoplasmosis at the laboratory, in HUSM. The positive or negative status of the samples was first determined by Elecsys® *Toxoplasma* IgG and IgM Immunoassays (Roche, Germany). Based on the serological profiles, serum samples were divided into four groups: Group I consisted of 151 anti-*Toxoplasma* IgG positive serum samples. Group II consisted of 96 IgG negative sera. Group III consisted of 17 sera from patient infected with diseases other than toxoplasmosis. Group IV consisted of 6 anti-*Toxoplasma* IgM positive sera. Additionally, 30 human serum samples from apparently healthy blood donors were collected and used as negative controls for the determination of the assay cut-off value.

### Samples size calculation

The sample size was calculated using PS software for single proportion formula and confirmed with sample size calculation for sensitivity & specificity studies designed by Dr. Mohd Ayub (Universiti Sains Malaysia) with the parameters indicated in Table 1. The desired sample number for Group I (151 IgG positive) and Group II (96 IgG negative) were successfully collected. Unfortunately only 6 anti-Toxoplasma IgM samples and 17 sera from patient infected with diseases other than toxoplasmosis were achieved during the study period. Due to the time limit the study was conducted with the collected serumsamples.

**Table 1:** Sample size calculation

Variable	p	d	Z	N
IgM+	0.95	0.06	95%	50
IgG+	0.83	0.06	95%	151
IgM- IgG-	0.90	0.06	95%	96
Other infection	0.95	0.06	95%	50

*N* sample size, *Z* Confidence level, *P* expected sensitivity and *d* precision

### Design, construction and expression of the recombinant multiepitope antigen

A single recombinant multiepitope antigen (USM.TOXO1) consisting of nine linear and conserved immunodominant within the SAG1, GRA2 and GRA7 antigens of *T. gondii* was designed as described previously [9]. Consequently, the corresponding gene encoding this antigen with final length of 435 bp was constructed by assembly PCR as described by Stemmer (1995) [23]. Two steps were involved in this assay: Gene assembly (1st PCR) and gene amplification (2nd PCR). For gene assembly, equal volume of 19 overlapping oligonucleotide was mixed to prepare the assembly mix (250 μM). The mixture was subsequently diluted 100 fold in 20 μl PCR mix containing 4 μl of 5X Phusion HF buffer, 0.4 μl of 10 mM dNTPs, and 0.2 Phusion Hot Start II DNA Polymerase (2 U/μl) (Thermo Scientific, USA). The mixture was then subjected to 98 °C for 30 s as initial denaturation, followed by 55 cycles of amplification at 95 °C for 1 min, 64 °C for 1 min, 72 °C for 1 min, and a final extension cycle at 72 °C for 10 min. In the gene amplification, two outside primers were designed to allow specific amplification of a desired gene from the collection of DNA fragment generated by assembly PCR, USM.TOXO1 forward primer (5’-ACGCGAATTCATGGGTCTCACGAGGACGTA-3′) and USM.TOXO1 reverse primer (5’-ACGTCAAGCTTCTATGGGCAGATTTGCCTG-3′).

The reaction was performed in final volume of 25 μl containing 5 μl of the first PCR product and 4 μl of 5× Phusion HF buffer, 1 μM each forward and reverse primers, 0.5 μl of 10 mM dNTPs, 0.25 μl Phusion Hot Start II DNA polymerase (2 U/μl) and sterile ddH_2_O were added to make the final volume of 25 μl. The PCR amplification was carried out under the following conditions: initial denaturation 98 °C for 30 s, followed by 23 cycles of amplification at 95 °C for 1 min, 64.5 °C for 1 min, 72 °C for 1 min and final extension 72 °C for 10 min. Subsequently, the USM.TOXO1 synthetic gene was cloned into pET-32a(+) expression vector (Novagen, U.S.A). Afterward, the protein expression was induced in *E. coli* expression system and the synthetic protein was successfully purified using Ni-NTA spin column as described previously [9].

### Development of in-house indirect-ELISA using USM.TOXO1 as capture antigen

Indirect ELISA was developed to detect the anti- IgG antibodies against recombinant USM.TOXO1 antigen. The optimal concentration of the coating antigen and the serum, conjugate dilution were determined by checkerboard titration assay using known positive and negative human sera. As the result, the concentration show highest discrimination value between positive and negative sera was considered to be optimal. After optimization, the ELISA was carried out using standard conditions. Briefly, a 96-well Microplates was coated with 100 μl of USM.TOXO1 recombinant antigen to the final concentration of 2.5 μg/ml in 0.05 M carbonate buffer (pH 9.6) and incubated overnight at 4 °C. The following day the wells were washed (3X) with PBS-T for 5 min each time and blocked with 200 μl of blocking buffer for 1 h at 37 °C. After another rounds of washing, 100 μl of human sera diluted at 1: 400 was added to the wells and incubated at 37 °C for 1 h. At the end of the incubation time the wells were washed again and 100 μl of HRP conjugated anti-human IgG antibody (diluted 1:4000) was added for 1 h at 37 °C, followed by final 3X wash.

The immunoenzymatic color reaction was developed by adding 100 μl of TMB substrate and the plate was further incubated for 15 min. Finally the reaction was stopped by adding 100 μl of 2 M H_2_SO_4_ and the optical density (OD) at 450 nm was then measured by using SpectraMax M Series Multi-Mode Microplate Readers (USA). The cut-off value was established as the average OD value of 30 serum samples from healthy negative control blood donor plus 3 standard deviations. Therefore, serum were considered negative or positive when its optical density less or more than adjusted cut off value respectively [24, 25].

### Determination the cross-reactivity of the USM.TOXO1 IgG ELISA

To determine the cross-reactivity of the USM.TOXO1 IgG ELISA, 17 serum samples obtained from patients with infections other than toxoplasmosis (Dengue; 2 samples, HBV; 1 sample, CMV; 1 sample, HSV; 7 samples, amoebiasis; 5 samples, ascariasis; 1 sample, *Salmonella* Typhi; 2 samples and *Shigella* spp.; 1 sample) were examined.

### Reactivity of the USM.TOXO1 with *T. gondii* IgM antibodies

Due to the small number of positive IgM samples (6 serum samples) obtained in this study. The immunoreactivity of USM.TOXO1 antigen against anti-*T.gondii* IgM antibodies was confirmed by western blot and Dot enzyme immunoassay (dot-EIA) analysis. The western blot analysis was performed similarly as described previously [9] with exception of using anti-human IgM conjugated with alkaline phosphatase. The color reaction was developed using alkaline phosphatase conjugate substrate. For the dot-EIA, a concentration of 0.6 mg/ml of USM.TOXO1 was dotted onto PVDF membrane. The membrane was allowed to dry at room temperature for 1 h. The blocking step, incubation with primary and secondary antibodies was performed as described for western blot.

### Statistics

The sensitivity, specificity, negative, and positive predicted values were calculated by MedCalc online Software, using the following formulas: sensitivity: TP/TP + FN × 100%; specificity: TN/TN + FP × 100%; positive predicted value: TP/TP + FP × 100%; negative predicted value: TN/TN + FN × 100, where TP is the number of true positive; FP, the number of false-positive; FN, the number of false negative; and TN, the number of true negative.

## Result

### Production of the USM.TOXO1 multiepitope antigen

**Fig. 1:**
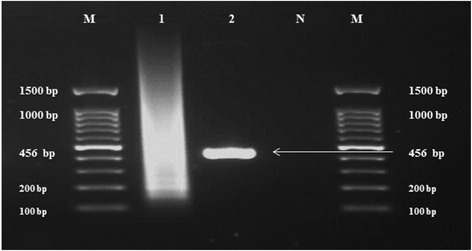
Construction of the USM.TOXO1 synthetic gene by assembly PCR. Lane M: 100 bp DNA Marker. Lane 1: 1st PCR product. Lane 2: Expected USM.TOXO1 gene (456 bp) amplified in the 2nd PCR. Lane N: Negative control

In this study, a single synthetic gene (456 bp) encoding nine immunodominat epitopes of *T. gondii* antigens was designed as previously described [9]. Subsequently the gene was successfully constructed and amplified by experimental methods using assembly PCR (Fig. 1). The corresponding recombinant multi-epitope protein was successfully expressed as and purified.

### Evaluation of diagnostic potential of the purified USM.TOXO1 recombinant proteins by indirect ELISA

To evaluate the potential of USM.TOXO1 antigen for the detection of anti *T. gondii* IgG antibodies in human sera, an in-house ELISA was developed using USM.TOXO1 fusion proteins as capture antigen. As shown in Table 2, 129 out of 151 positive sera (group I) were reacted with USM.TOXO1 antigen with ODs above the cut-off value, whereas, the generated ELISA failed to detect *T. gondii* specific antibodies in 22 positive sera, resulting in a sensitivity of 85.43%. The USM.TOXO1 ELISA was negative in 78 out of 96 samples from group II (negative serum samples), while 18 samples showed false positive results with OD_450_ values higher than the cut-off value yielding a specificity of 81.25%. The positive and negative predictive values of the generated ELISA were 87.76% and 78% respectively (Table 3).

**Table 2:** Comparison of Roche Elecsys commercial ELISA and USM.TOXO1 ELISA for the detection of the anti-*T. gondii* IgG antibodies in patients sera

USM.TOXO1 ELISA				
		Positive, n (%)	Negative, n (%)	Total
Commercial ELISA	Positive, n (%)	129 (87.75)	22 (22)	151
	Negative, n (%)	18 (12.25)	78 (78)	96
	Total	147	100	247

**Table 3:** Sensitivity and specificity of the USM.TOXO1 ELISA for the detection of anti-*T. gondii* IgG antibody

Sensitivity (%) (95% CI)	Specificity (%) (95% CI)	Positive predictive Value (%) (95% CI)	Negative Predictive Value (%) (95% CI)
85.43 (78.78 to 90.64)	81.25 (72.00 to 88.49)	87.76 (81.34 to 92.58)	78.00 (68.61 to 85.67)

### Determination the cross-reactivity of the USM.TOXO1 IgG ELISA

The cross-reactivity of the USM.TOXO1 IgG ELISA indicated in Table (2) showed that 14 out of 17 sera were negative, while 3 samples generated false positive results.

### Reactivity of T. gondii IgM antibody in immunoblots with USM.TOXO1

**Fig. 2:**
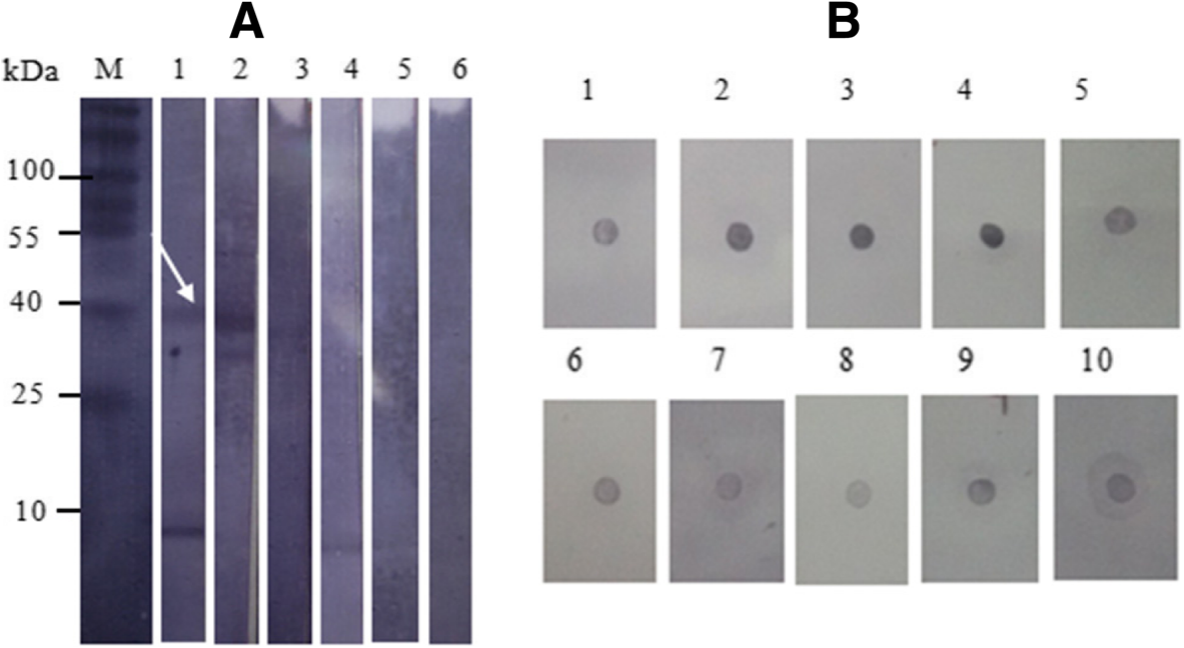
Reactivity of USM.TOXO1 with *T. gondii* IgM antibody by using immunoblots*.* **a** Western blot: Lane M: Molecular weight marker, Strips 1–3: Results of 3 positive sera, Strips 4–5: Results of negative sera **b** Dot-EIA: Strips 1–5: Results of positive sera, Strips 6–10: Results of negative sera

The immune reactivity of USM.TOXO1 antigen against anti-*toxoplasma* IgM antibody was evaluated by western blot and dot enzyme assay only. The results indicated in Fig. 2, demonstrate that USM.TOXO1 has the potential to detect toxoplasmosis-specific IgM antibody.

## Discussion

The serological tests play a vital role in the diagnosis of both human and animal toxoplasmosis [6]. Thus, researchers continue to strive in perfecting and improving the serodiagnostics of *T. gondii* infections. In this regard, acquiring effective diagnostic antigens would be highly beneficial. The current immunoassays are mainly based on the *T. gondii* lysate antigens (TLAs), which are characterized as high sensitive and specific diagnostic tools [26].

However, the insufficient accuracy of some diagnostic tests are correlated with significant variation in the procedure of producing such kind of antigens, resulting in a major drawback which is lack of the standardization. The real challenge for researchers is to identify novel antigens that possess high immunoreactivity [27]. Thus, exploration of effective diagnostic reagents is the best strategy for the development of accurate diagnostic assays, which would considerably improve the management of the disease [16]. Accordingly, significant efforts have been exerted and thousands of studies have been conducted. Studies on the development of standard diagnostic markers usually assume that developing a single antigen expressing immunodominant regions for all parasite life stages would greatly improve *T. gondii* diagnostic strategies [8]. The peptide-based antigens appears as attractive and promising antigenic candidates for the achievement of standard diagnostic marker [28].

At present, bioinformatics tools play a significant role in the identification of immunodominant epitopes [29]. Meanwhile, the advancement of molecular techniques allows the production of recombinant multiepitope antigen [30]. Interestingly, the uses of epitope-based antigens could allow better standardization of the diagnostic tests [21]. Furthermore, the diagnostic value of a particular epitope can be studied; thus, the sensitivity of the immunoassays may be enhanced by combining several epitope antigens [8, 21]. Compared with the lysate antigens, epitope-based antigens exhibit several advantages in the serological investigation of toxoplasmosis. These benefits include the low cost of the production and purification protocol, the precise knowledge on the composition of the diagnostic antigen, and the ability to use multiple epitopes that represent different stages of the infection [28].

Until now, only a few studies have demonstrated the usefulness of the recombinant multiepitope antigens in the detection of anti-*T. gondii* antibodies in human sera [8, 18, 21, 28]. In the present study, this concept was tested. This study speculated that developing a novel recombinant antigen expressing the potential immunodominant epitopes of three *T. gondii* antigens would be an effective strategy to improve the sensitivity and specificity of the diagnostic assays. Accordingly, USM.TOXO1 gene was designed and successfully constructed by assembly PCR. Compared with the previous studies various methods have been developed to produce multi epitope-based antigens [8, 18, 21, 28], however, assembly PCR is inexpensive as well as more practical strategy for constructing synthetic genes encoding different epitopes or more than one copies of the same epitope. Following the production, the potential uses of the USM.TOXO1 as diagnostic marker was examined. An indirect IgG (ELISA) was developed to detect anti-*T. gondii* antibodies in human sera.

The results indicated that USM.TOXO1 represents a valid and promising diagnostic marker for screening of anti-*T. gondii* in human sera. The USM.TOXO1 ELISA specifically identified 129 out of 151 serum samples from the sero-positive *T. gondii* patients. Meanwhile, 18 serum samples from the sero-negative patients showed false positive results. The diagnostic performance observed for the new antigens developed in this work was achieved at the maximum values of 85.43%, 81.25%, 87.76%, and 78% for diagnostic sensitivity, specificity, positive predictive value (PPV), and negative predictive value (NPV), respectively. These findings are compatible with the diagnostic performance of several recombinant antigens developed recently for the diagnosis of toxoplasmosis [3, 16]. However, in terms of cost and efficiency, the protocol performed in this study is less expensive and can rapidly produce large quantities of the recombinant protein.

The results emphasized the usefulness of the USM.TOXO1 ELISA in the serological screening of toxoplasmosis. This notion is supported by the high sensitivities and specificities, which exceeded 85% and 80% respectively. However, the values did not exceed 90% even though USM.TOXO1 containing the most antigenic epitopes of SAG1, GRA2, and GRA7. This might be due to the loss of antigenicity due to the incorrect folding of the recombinant proteins expressed in the *E. coli* expression systems [6]. Thus, some of the epitopes featured in the native antigen may not have been presented in the recombinant protein and therefore, cannot be recognized by *T. gondii* or cross-react with other antibodies. Additionally, the epitope’s diagnostic value could also be affected by immune diversity, which is a major hurdle that prevents the achievement of the high epitope predicted diagnostic value [31].

The sensitivity of the developed ELISA was similar to that found by Dai et al. (2012), in which a recombinant multiepitope peptide (rMEP) was developed to express three antigenic determinants of SAG1, SAG2, and SAG3 antigens. However, the specificity was much lower in the current study than the 100% obtained by Dai et al. (2012). The data obtained from a study conducted by Faria et al. (2015) also showed promising results, in which recombinant multiepitope proteins reacted with 88.8% of the positive sera and provided a specificity of 80%. Such result is consistent with our findings. However, multiepitope antigens were highly sensitive and specific in detecting anti-*Trypanosoma cruzi* antibody and specifically differentiating *P. vivax* from *P. falciparum* infection, suggesting powerful tools for developing accurate diagnostic assays [31, 32]. Furthermore, a sensitivity and specificity of 100% was also reported [33].

The current paradigm strongly supports the further development of peptide-based assays. Such assays would benefit the diagnosis because of the variation in the host humoral immune response from stage-specific immunity. The response variation would produce specific IgG antibodies associated with one stage of infection but not with the other stages. Thus, multiepitope antigens, which express various antibody-binding sites from different antigens in different stages of infection must be used to develop diagnostic assays that can detect a wide range of antibodies produced throughout the disease process [6].

The future prospective in the establishment of an effective serodiagnostic assay for the detection of *T. gondii* infection should focus on identifying novel antigenic determinants along with examining various cocktails of distinct epitope-based antigens. The goal is to attain an appropriate level of sensitivity and specificity that would be unaffected by antigenic variation and give accurate results.

The rational selection of the *T. gondii* antigens that possess conserved T and B cell epitopes is crucial for the successful application of this epitope-based strategy [34]. Thus, SAG1, GRA2, and GRA7 have been selected as the candidate antigens to be assessed in the current project. All of these antigens have been the subject of various fundamental studies. The findings of most of these studies demonstrated the potential of these antigens to become more successful diagnostic reagents or/and effective vaccines.

SAG1 is of particular interest because it represents around 5% of the tachyzoite antigen [35]. Investigations on the immunogenicity and immunoreactivity of SAG1 repeatedly yielded significant results [36, 37]. These reasons explain the selection of SAG1 as an antigen candidate in this study. Previous studies indicated that GRA7 is a promising vaccine candidate and novel diagnostic reagent [38]. Direct contact of GRA7 with the host immune system enhances the induction of strong antibody and cell-mediated responses in both acute and chronic infection [5].

Similar to SAG1 and GRA7, GRA2 is also characterized as a highly immunogenic antigen during *T. gondii* infections; it has the potential to induce protective immune response in both human and experimental models [39]. GRA2 allowed the differential identification of anti-toxoplasma antibody in acute and chronic human infections [40]. These data suggest that SAG1, GRA7, and GRA2 antigens could advance the development of effective diagnostic reagents for *T. gondii*.

## Conclusion

In conclusion, the diagnostic performance of synthetic protein expressing nine epitope of *T. gondii* was evaluated. The results indicate that this antigen might be a promising epitope based antigen for seodiagnosis of *T. gondii* infection which can be modified later to improve the sensitivity and the specificity by either increase the number or the types of epitopes or manipulate the protein structure. There are two main limitations in this study; where only B cell prediction software was applied, in an ideal situation, the T cell prediction epitope should also be considered. Another limitation is the performance of USM.TOXO1 in IgM ELISA was not tested, due to the limited IgM positive sera collected during the study period.

## Abbreviations

CMV: Cytomegalovirus
Dot-EIA: Dot enzyme immunoassay analysis
ELISA: Enzyme-linked Immunosorbent Assay
GRA: Dense granule antigen
HSV: Herpes simplex virus
OD: Optical density
PCR: Polymerase chain reaction
SAG1: Surface antigen1
